# Dapagliflozin attenuates cisplatin-induced nephrotoxicity in rats through modulation of ROS/NF-κB, BCL2/Bax and PINK1/Parkin signaling pathways

**DOI:** 10.1038/s41598-026-50755-0

**Published:** 2026-05-17

**Authors:** Esraa K. Khallaf, Eman A. Ramadan, Mohey M. Elmazar, Marwa M. Safar

**Affiliations:** 1 Department of Pharmacology and Biochemistry, Faculty of Pharmacy, The British University in Egypt, Cairo, Egypt; 2 Department of Pharmacology and Toxicology, Faculty of Pharmacy, Cairo University, Cairo, Egypt

**Keywords:** Dapagliflozin, Cisplatin, Acute kidney injury (AKI), Sodium-glucose cotransporter-2 (SGLT-2) inhibitors, Apoptosis, Mitophagy, Biochemistry, Diseases, Drug discovery, Nephrology

## Abstract

Dapagliflozin (DPG), an anti-diabetic drug, has gained attention for its renal protective effects through multiple molecular pathways, yet its impact on mitophagy in cisplatin (CIS) nephrotoxicity remains unclear. This study aimed to examine the impact of DPG against CIS-induced nephrotoxicity in rats, targeting mainly PINK1/Parkin-mediated mitophagy and inflammatory/apoptotic pathways. Male Sprague Dawley rats received DPG (10 mg/kg; p.o) daily for 14 consecutive days and AKI was induced by a single injection of CIS (7 mg/kg; i.p) on day 10. Blood glucose, serum levels of creatinine and urea nitrogen, oxidative stress, inflammatory, apoptotic, mitophagy markers, and histological changes were assessed. DPG reduced glomerular and tubular damage by alleviating NGAL and KIM-1 protein expression as well as MDA and NO accompanied by enhanced GSH expression. It mitigated gene expression of NF-κB, TNF-α and IL-6 along with downregulation of Bax and upregulation of BCL2 mRNA expression. DPG prevented apoptotic activity through reduction in cleaved caspase-3 immunoreactivity. Moreover, DPG restored CIS-mediated mitophagy inhibition evidenced by elevation of PINK1, Parkin and LC3II/LC3I ratio and reduction of TIMM23, TOMM20 and p62. In conclusion, DPG prevents CIS nephrotoxicity probably, *via* activating PINK1/Parkin, meanwhile attenuating oxidative stress and apoptotic activity.

## Introduction

Acute kidney injury (AKI) is a major public health problem associated with a rapid deterioration of kidney function along with a decline in urine output and a marked elevation in serum creatinine (SCr)^1^. AKI is critically related to the development of short adverse events such as electrolyte imbalance, fluid overload, inflammatory response, and long-term events such as chronic kidney disease and end-stage kidney disease^2^. Epidemiological studies reported that the annual incidence of AKI is estimated to be 13.3 million with mortality rate up to 1.7 million^3^. Regardless of economic situation, AKI is a common life-threatening syndrome affecting 5% to 7.5% of inpatients and up to 50% to 60% of the intensive care unit patients^4^.

Cisplatin (CIS), an inorganic platinum-based chemotherapeutic agent, is a potent anti-neoplastic drug widely applied in the management of varieties of solid malignant tumors alone or as a tailored combination therapy^5^. In spite of CIS effectiveness in treating many solid-state cancer, its therapeutic use is frequently constrained by various significant undesirable effects including neurotoxicity, ototoxicity, hepatotoxicity, and nephrotoxicity which is the major dose-limiting side effect^6^. Approximately 30%−40% of patients who take CIS experience renal dysfunction^7^. Administration of standard doses of CIS (50–120 mg/m^2^) causes different degrees of nephrotoxicity through damage of proximal tubular cells, vascular injury**,** disruption of redox status and inflammatory response^8^. The initiation of nephrotoxic effects is mainly related to CIS cellular uptake and accumulation into proximal tubular epithelial cells^7^. Proximal tubular cells selectively exhibit high concentration of CIS compared to that in serum due to upregulation of organic cation transporters as well as copper transporter 1^9^. Inevitably, CIS is rapidly hydrolyzed to positively charged hydrating molecule causing DNA damage, mitochondrial dysfunction and cell death^10^. In proximal tubular cells, CIS mediate renal tubular damage through accumulation in negatively charged mitochondria causing mitochondrial dysfunction, disturbance of redox balance and so loss of mitochondrial depolarization^7^.

Autophagy is a cellular process of “self-eating” in which a cell removes misfolded or aggregated proteins and recycles damaged organelles, especially during periods of stress or starvation for cellular homeostasis^11,12^. Mitophagy is a form of autophagy that has a protective role in the removal of dysfunctional mitochondria during conditions of metabolic stress *via* Parkin-dependent and/or Parkin-independent pathways^13^. In Parkin-dependent pathway, removal of damaged mitochondria occurs through the activation of parkin, the ubiquitination of mitochondrial proteins and the recruitment of autophagy adaptors such as optineurin and sequestosome 1 (p62/SQSTM1) which interact with LC3^14^. Unlike Parkin-dependent pathway, mitochondrial outer membrane proteins such as BCL2 interacting protein 3 (BNIP3) and FUN14 domain containing 1(FUNDC1) directly bind with LC3 promoting sequestration of targeted mitochondria into autophagosome and mitophagy activation^15^. It is known that the kidney exhibits the highest mitochondrial density second to the heart due to high energy demand. Therefore, impaired mitochondrial homeostasis is a key pathological feature in the development of AKI^14^. Emerging evidence suggests the potential role of both mitophagy pathways in CIS nephrotoxicity. Even so, Parkin-dependent pathway has been closely associated with the mitophagy activation in kidney diseases, whereas Parkin-independent pathway are poorly defined^16,17^.

Dapagliflozin (DPG), sodium-glucose cotransporter-2 (SGLT-2) inhibitor, has been validated by the FDA for the management of type 2 diabetes mellitus^18^. Literature reports have found that DPG offers cardioprotective and renoprotective benefits beyond diabetic control^19,20^. Lately, DPG has attracted considerable interest in multiple kidney injury models *via* exerting anti-inflammatory, antioxidant and anti-apoptotic effects. The role of DPG in regulating oxidative, inflammatory, apoptotic and fibrotic processes were previously delineated in a rat model of fructose-streptozotocin-induced diabetes^21^. DPG treatment in gentamicin-induced nephrotoxicity was associated with reducing renal tubular cell death through modulation of miR-21 and MiR-181^22^. DPG exhibited a potential role as autophagy regulator in diabetic nephropathy model through modulation of AMPK/mTOR signaling pathway^23^. Notably, DPG treatment has been found to protect against mitochondrial damage in models of myocardial ischemia reperfusion (IR) injury and diabetic nephropathy *via* modulation of Parkin dependent mitophagy pathway^24,25^. Interestingly, the use of DPG alone or in combination with silymarin was found to reduce oxidative stress and suppress inflammation in a rat model of CIS-induced nephrotoxicity^26^. However, the modulatory actions of DPG, particularly mitophagy pathway, in CIS-induced AKI are yet to be verified.

Understanding the underlying molecular mechanisms through which DPG exerts its protective effect, is crucial to pave the way for new mechanistic insights for AKI mitigation and prevention. Therefore, the present study was performed to investigate the possible protective effect of DPG in CIS-treated rats, pointing to its potential role as regulator of oxidative stress, inflammation, apoptosis, autophagy and mitophagy.

## Materials and methods

### Drugs and chemicals

CIS vial (1mg/ml) was purchased from Mylan Pharmaceuticals company (Saint-Priest, France). DPG was obtained from EVA Pharma (Cairo, Egypt).

### Animals

Sprague Dawley male rats, 6–8 weeks and weighing 150–250 g, were obtained from the animal house of the Faculty of Pharmacy, the British University in Egypt (Cairo, Egypt). Before model induction, animals were allowed to acclimatize one week. Rats were kept in three metabolic cages, under constant conditions of light/dark cycles, humidity (60 ± 10%) and constant temperature (23 ± 2 °C). They were fed a standard pellet diet (Meladco company, Obour City, Egypt) and allowed free access to water. All procedures were conducted following the standards of the Research Ethics Committee of the Faculty of Pharmacy, the British University in Egypt, Cairo, Egypt (EX-2318).

### Experimental design of CIS-induced nephrotoxicity

Nephrotoxicity was triggered by a single injection of CIS (7mg/kg; i.p) on day 10. The dose was selected as previously reported^27^**,** and supported by a pilot experimental trial. Eighteen rats were randomly divided into 3 groups (n=6) as follows: normal control (NC) group: received the vehicle of DPG (1% tween in 0.9% normal saline) for 14 days. CIS- treated group: received the vehicle of DPG for two weeks and a single injection of CIS at (7 mg/kg; i.p) on day 10. CIS+ DPG treated group: received DPG once daily at (10 mg/kg; p.o) for a period of 14 days^22^; and CIS at (7 mg/kg; i.p) was given once on day 10. Blood glucose level was recorded for all rats on day 1 and 14 using commercial blood glucose test strips.

### Sample collection and preparation

After 14 days of treatment, rats were anaesthetized with isoflurane using a vaporizer system. Blood was collected from eye retro-orbital sinus of the rats and then centrifuged for 15 min at 3000 rpm for serum separation and so evaluation of SCr and urea nitrogen. Subsequently, rats were euthanized by CO_2_ to harvest kidneys. Kidney specimens were quickly removed and kept at −80 °C for other molecular assessments except for three right kidneys of each group were instantly preserved in formalin/water (10%) for histopathological and immunohistopathological examination. Kidney samples were homogenised using phosphate buffer (pH 7.4) as 10% weight/volume then protein concentration was measured using the Pierce™ BCA Protein Assay Kit (cat#23,225) (Thermo Fisher Scientific, USA), following the manufacturer’s protocol.

### Biochemical examination

#### Renal function assessment

Renal function markers such as SCr (cat#235 001) and urea nitrogen (cat#318 001) levels were assessed colorimetrically with commercial kits supplied by (Spectrum, Cairo, Egypt), adhering to the manufacturers’ methods.

#### Enzyme-linked immunosorbent assay (ELISA)

Quantitative detection of neutrophil gelatinase-associated lipocalin (NGAL) (cat# MBS260195), Parkin (cat# MBS722554), kidney injury molecule-1 (KIM-1) (cat# MBS355395), PTEN-induced kinase 1 (PINK1) (cat# MBS9343426), microtubule-associated protein 1 light chain 3 (LC3)-I (cat#MBS3806505) and p62/SQSTM1 (cat# MBS7210079) were performed using ELISA kits (MyBioSource, USA). Renal levels of glutathione (GSH) (cat# GR 25 11), nitric oxide (NO) (cat# NO 25 33), and malondialdehyde (MDA) (cat# MD 25 29) were also assessed using rat ELISA kits (Biodiagnostic, Giza, Egypt). Besides, translocase of inner mitochondrial membrane 23 (TIMM23) (cat# ABIN1744533) and translocase of outer mitochondrial membrane 20 (TOMM20) (cat# ABIN1744540) were measured by rat ELISA kits (Antibodies,USA). Finally, CELLBIOLABS ELISA kit was used to measure LC3-II (cat# CBA-5116) level. All procedures were done following the manufacturers’ instructions.

#### Quantitative real-time reverse transcription polymerase chain reaction (qRT-PCR)

Total RNA from kidney tissue homogenate was isolated using GeneJET RNA Purification kit (cat#K0732) (Thermo Fisher Scientific, USA). Thereafter, reverse RNA transcription was performed using High-Capacity cDNA Reverse Transcription Kit (cat#4,368,813) (Thermo Fisher Scientific, USA). The Nanodrop One spectrophotometer (Thermo Fisher Scientific, USA) was used to measure concentration and purity of the RNA. Maxima SYBR Green qPCR master mix (cat#K0221) (Thermo Fisher Scientific, USA) with StepOne™ Real-Time PCR System (Software version 3.1, Applied Biosystem, USA) was utilized for synthesis of cDNA and PCR amplification protocol. All experimental procedures were performed based on the manufacturers’ instructions. The primer sequences were designed using the online NCBI primer tool (https://www.ncbi.nlm.nih.gov/tools/primer-blast/) and provided by (Thermo Fisher Scientific, USA) (Table 1). The relative expression of mRNA levels of inflammatory markers: nuclear factor kappa B p65 (NF-κB p65), interleukin (IL)−6 and tumor necrosis factor alpha (TNF-α), in addition to apoptotic markers: Bcl-2-associated protein x (Bax) and B-cell lymphoma 2 (BCL2) was determined using 2^^-ΔΔCt^ method^28^.

**Table 1:** The primer sequences for qRT-PCR.

Gene	Forward primer (5′−3′)	Reverse primer (3′−5′)
NF-κB p65	CAAGCCATTAGCCAGCGCAT	TGTTGGGGGCACGGTTATCA
IL-6	AGCCACTGCCTTCCCTACTTC	GACAGTGCATCATCGCTGTTCAT
TNF-α	CTTCTCATTCCTGCTCGTGG	TGATCTGAGTGTGAGGGTCTG
Bax	GACGGCAACTTCAACTGGGG	GAAAGGAGGCCATCCCAGCC
BCL2	AAGCACATCCAATAAAAGCGC	GTTATCATACCCTGTTCTCCCG
β-actin	CCACCCGCGAGTACAACCTT	CCCACGATGGAGGGGAAGAC

### Histopathology

Kidney tissues were rapidly immersed in 10% (v/v) formalin and washed in xylene then dehydrated in a serial series of ethanol. After dehydration, tissue samples were embedded in paraffin wax and cut into 4-µm slices with a rotary microtome (microTEC, Duisburg, Germany) for haematoxylin and eosin (H&E) staining. Under light microscope (CX 41RE, Olympus Corporation, Tokyo, Japan) at a magnification of X400 (Olympus soft imaging, SC100, Germany), eight random fields were captured for each group. The degree of morphological changes was evaluated by a pathologist in a blinded manner using predefined scoring system^29^.

### Immunohistochemistry

For immune staining of cleaved caspase-3 and p62, the avidin–biotin peroxidase complex method was used^30^. Briefly, paraffin embedded tissue sections were deparaffinized and rehydrated; thereafter, an antigen heat retrieval step was carried out in a microwave oven in citrate buffer. After blocking, the primary monoclonal antibodies against cleaved caspase-3 (cat# GB11532) (ServiceBio, China) and p62 (cat# bs-2935R) (Bioss, USA) (at a dilution of 1:100) were incubated 30 min with the tissue sections for immunostaining. After washing, secondary antibody was applied for 20 min. Sections were washed and immunoreactivity was detected using 4,6-diamidino-2-phenylindole (DAPI) mixture, a nuclear fluorescent stain, for 10 min. Following this, Harris’s haematoxylin was used to counterstain sections. Stained slides were imaged by light microscopy (CX 41RE, Olympus Corporation, Tokyo, Japan), and fluorescence intensity of the target protein was evaluated in eight randoms fields per group using ImageJ software (NIH, USA). Analysis of immunohistochemistry (IHC) was carried out by a pathologist blinded to the experimental design.

### Statistical analysis

Data were expressed as the mean ± S.D. Statistical analysis were attained using one-way analysis of variance (ANOVA), followed by Tukey–Kramer multiple comparison test to ascertain differences between groups. P values < 0.05 were regarded statistically significant. For the blood glucose, two-way ANOVA test was performed. All statistical analyses were done using GraphPad Prism nine software (GraphPad Software Inc., USA).

## Results

### Effects of dapagliflozin pretreatment on kidney function and AKI early biomarkers in cisplatin-treated rats

**Fig. 1:**
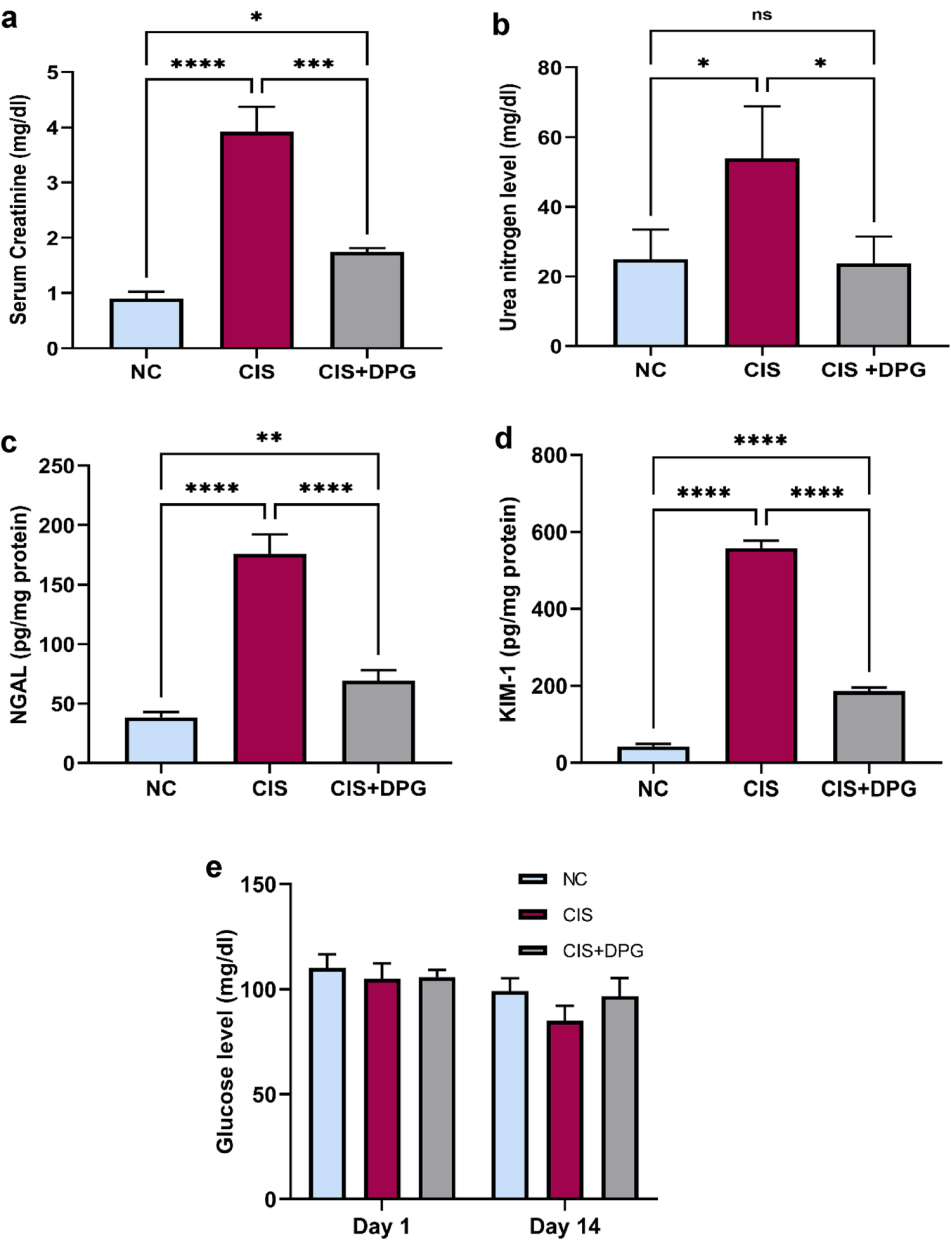
Effects of dapagliflozin pretreatment on kidney function markers of cisplatin-treated rats. Blood samples were obtained, and (**a**) Serum creatinine (SCr) and (**b**) Urea were measured. Tissue content of (**c**) Neutrophil gelatinase-associated lipocalin (NGAL) and (**d**) Kidney injury molecule-1 (KIM-1) were measured in the whole kidney lysate using ELISA. (**e**) Non-fasting blood glucose level was measured at day 1 and day 14 using two-way ANOVA test. All the data were represented as mean ± SD (n = 6). Normal control, NC; Cisplatin, CIS; and Cisplatin + Dapagliflozin, CIS + DPG groups. Statistical analysis was attained using one-way analysis of variance (ANOVA), followed by Tukey–Kramer multiple comparison; non-significant difference (ns), *p < 0.05, **p < 0.01, ***p < 0.001, ****p < 0.0001.

Administration of CIS (7 mg/kg) clearly elevated SCr and urea nitrogen levels by 4-and 2-fold, respectively, compared to NC group, suggesting the occurrence of renal injury. In the present study, rats pretreated with DPG (10 mg/kg) showed a marked reduction in SCr and urea nitrogen by 55% and 56%, respectively, compared to CIS-treated rats (Fig. 1a,b). Consistent with these changes, NGAL and KIM-1 protein expression were significantly elevated following CIS administration by 5- and 13-fold, respectively, whereas DPG pretreated rats showed a marked reduction of theses markers by 61% and 67%, respectively, (Fig. 1c,d). It is noteworthy that there were no significant changes in non-fasting blood glucose level in all experimental groups following DPG and CIS treatment (Fig. 1e).

### Effects of dapagliflozin pretreatment on oxidative stress markers in cisplatin-treated rats

**Fig. 2:**
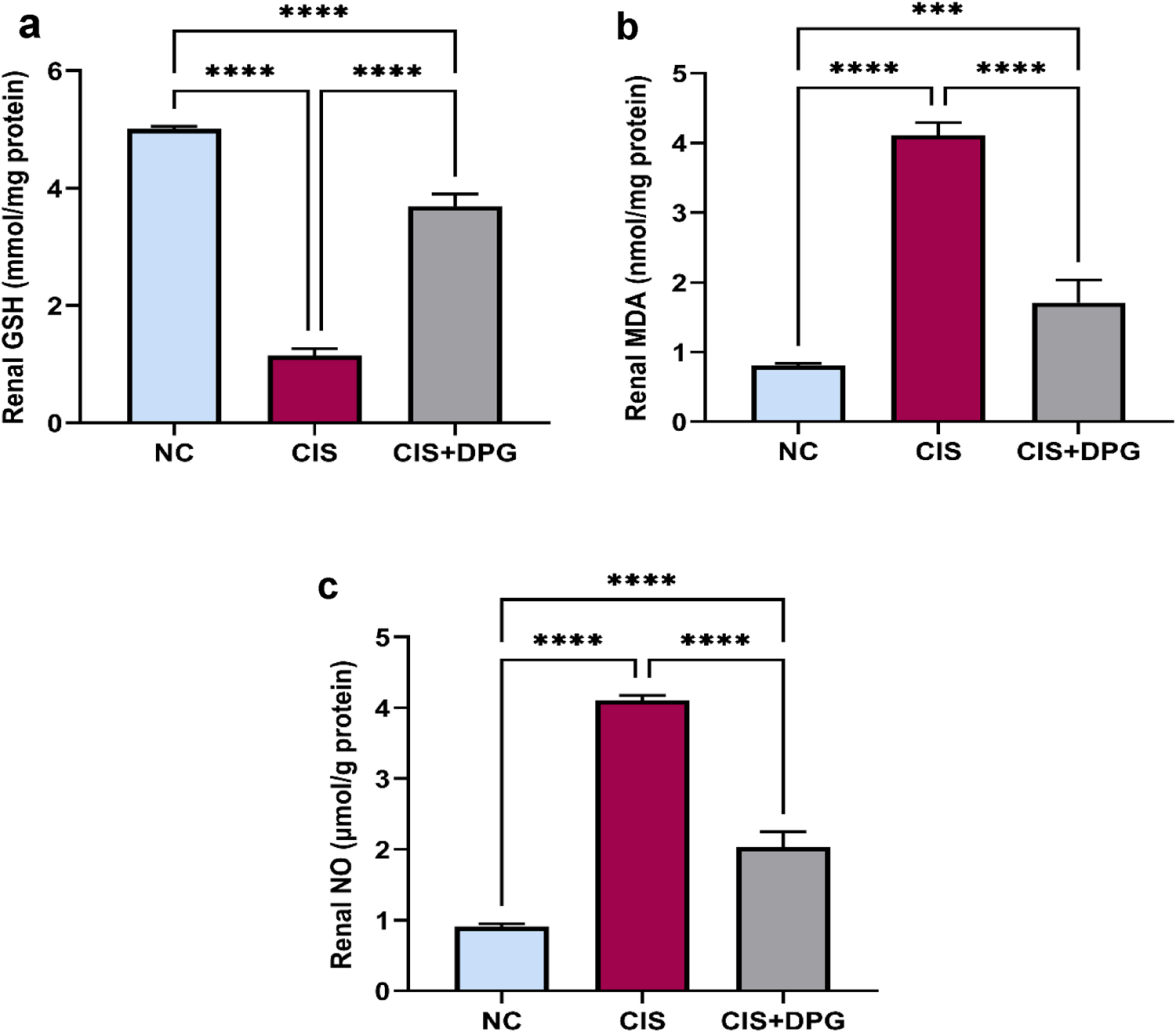
Effects of dapagliflozin pretreatment on oxidative stress markers in cisplatin-treated rats. The protein expression of (**a**) Glutathione (GSH), (**b**) Malondialdehyde (MDA), and (**c**) Nitric oxide (NO) in kidney tissues of rats were measured using ELISA. All the data were represented as mean ± SD (n = 6). Normal control, NC; Cisplatin, CIS; and Cisplatin + Dapagliflozin, CIS + DPG groups. Statistical analysis was attained using one-way analysis of variance (ANOVA), followed by Tukey–Kramer multiple comparison; ***p < 0.001, ****p < 0.0001).

CIS insult promoted disruption of redox status, as verified by a significant decline in renal GSH by 4-fold, alongside a significant increase in MDA and NO by 5-fold as compared to NC rats. While pretreatment with DPG showed a significant increase in renal GSH by 69%, together with a notable decrease in MDA and NO expression by 58% and 50%, respectively, compared to CIS-treated rats (Fig. 2a–c).

### Effects of dapagliflozin pretreatment on inflammatory markers in cisplatin-treated rats

**Fig. 3:**
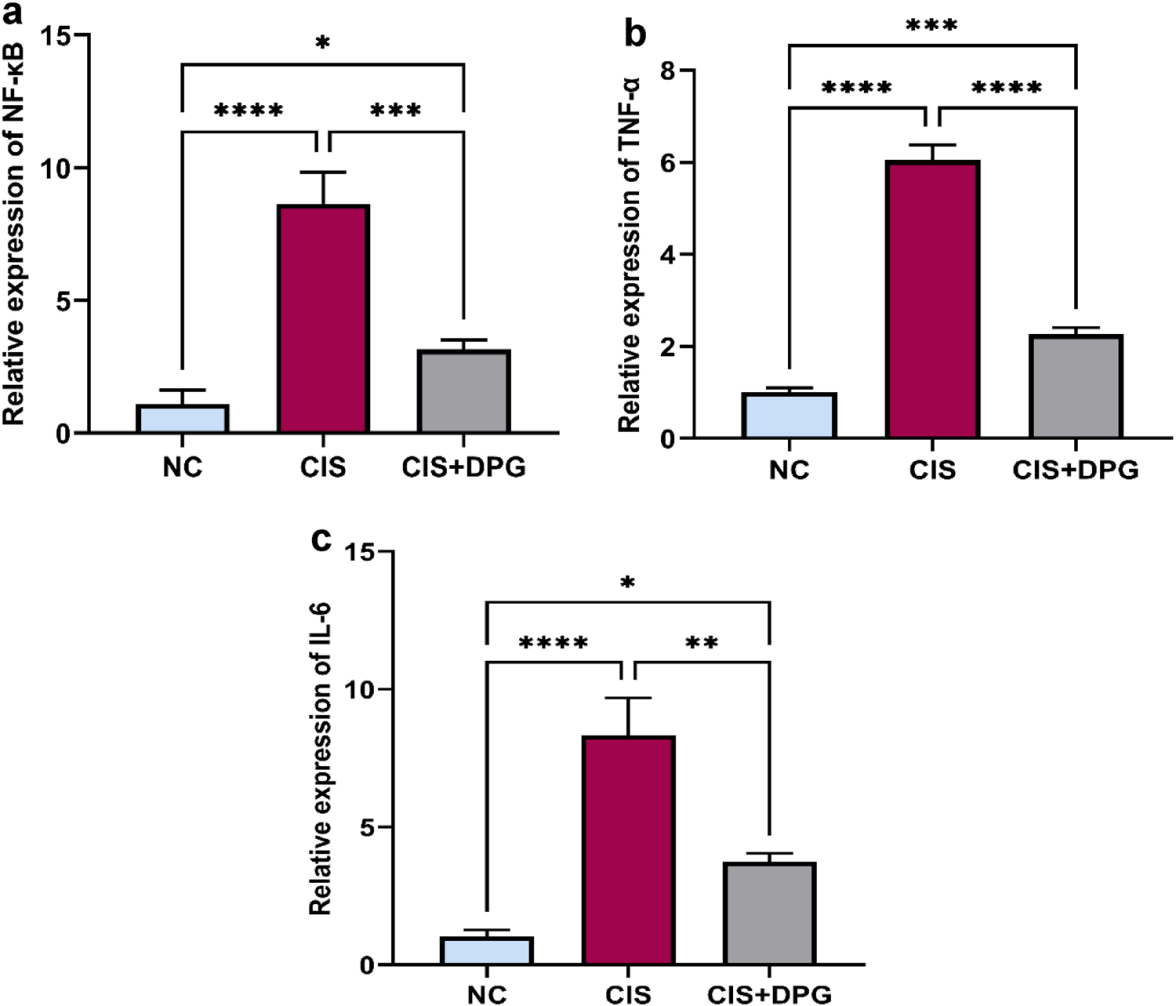
Effects of dapagliflozin pretreatment on inflammatory markers in cisplatin-treated rats. Quantification of mRNA expression levels of (**a**) Nuclear factor kappa B (NF-κB), (**b**) Tumor necrosis factor alpha (TNF-α), and (**c**) Interleukin-6 (IL-6) in rat kidneys. All the data were represented as mean ± SD (n = 6). Normal control, NC; Cisplatin, CIS; and Cisplatin + Dapagliflozin, CIS + DPG groups. Statistical analysis was attained using one-way analysis of variance (ANOVA), followed by Tukey–Kramer multiple comparison; *p < 0.05, **p < 0.01, ***p < 0.001, ****p < 0.0001).

CIS induced renal damage caused a prominent elevation of NF-κB p65 mRNA levels by 8-fold, leading to upregulation of mRNA levels of TNF-α and IL-6 by 6- and 8-fold, respectively, as compared to NC rats. Pretreatment with DPG resulted in a significant suppression of NF-κB p65 nuclear translocation by 63% as compared to CIS-treated rats. Sequentially, mRNA levels TNF-α and IL-6 were downregulated by 63% and 55%, respectively, compared to CIS-treated rats (Fig. 3a–c).

### Effects of dapagliflozin pretreatment on apoptotic markers in cisplatin-treated rats

**Fig. 4:**
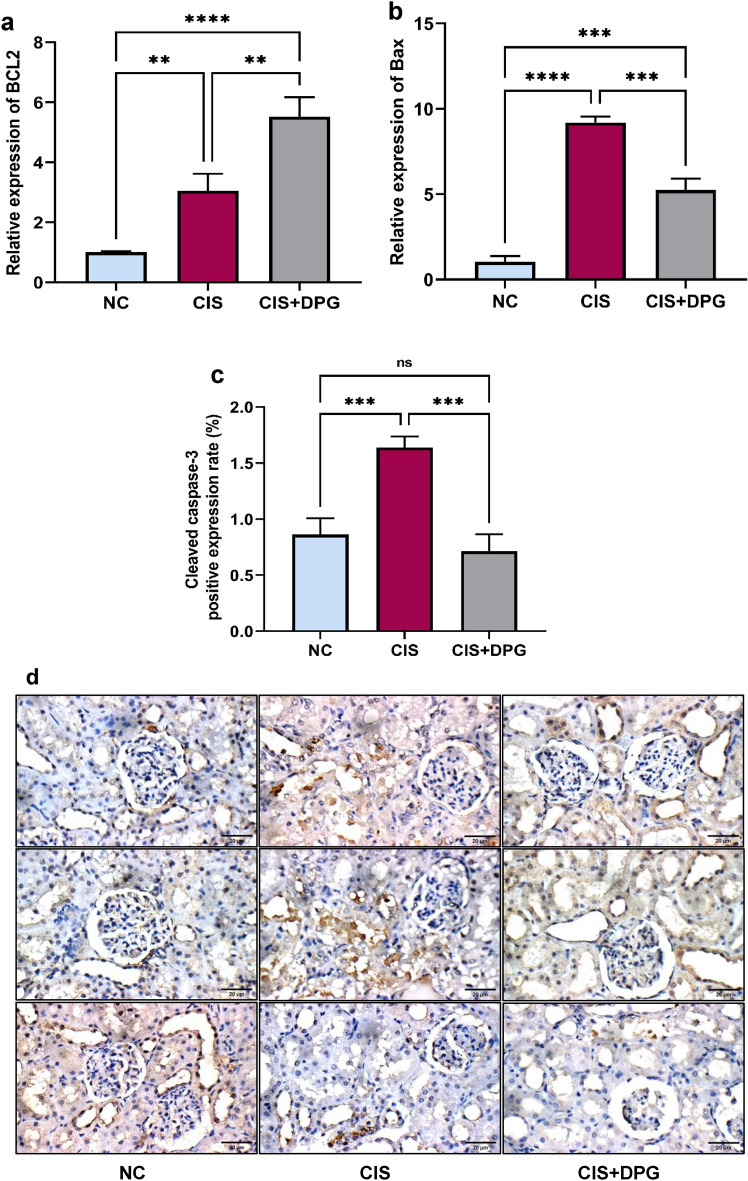
Effects of dapagliflozin pretreatment on apoptotic markers in cisplatin-treated rats. Quantification of mRNA expression levels of (**a**) B-cell lymphoma 2 (BCL2), and (**b**) Bcl-2-associated protein x (Bax) in rat kidneys. (**c**) Quantification of immunostaining of Cleaved caspase-3 from the images in (**d**) (× 400), scale bar = 20 µm. All the data were represented as mean ± SD (n = 6). Normal control, NC; Cisplatin, CIS; and Cisplatin + Dapagliflozin, CIS + DPG groups. Statistical analysis was attained using one-way analysis of variance (ANOVA), followed by Tukey–Kramer multiple comparison; non-significant difference (ns), **p < 0.01, ***p < 0.001, ****p < 0.0001).

CIS administration promoted apoptosis as demonstrated by a significant elevation of Bax mRNA level by 9-fold as compared to NC rats. On the other hand, CIS-treated rats markedly increased BCL2 mRNA levels by 5-fold compared to NC rats. Pretreatment with DPG remarkably upregulated BCL2 mRNA expression by 50% and downregulated Bax gene expression by 43% compared to CIS-treated rats (Fig. 4a,b). Additionally, immunohistochemical examination of kidney tissue exhibited a notable increase in cleaved caspase-3 immunoreactivity to approximately 2-fold in CIS-treated rats compared to NC rats, while DPG pretreatment significantly mitigated such elevation by 57% as compared to CIS rats (Fig. 4c,d).

### Effects of dapagliflozin pretreatment on mitophagy markers in cisplatin-treated rats

**Fig. 5:**
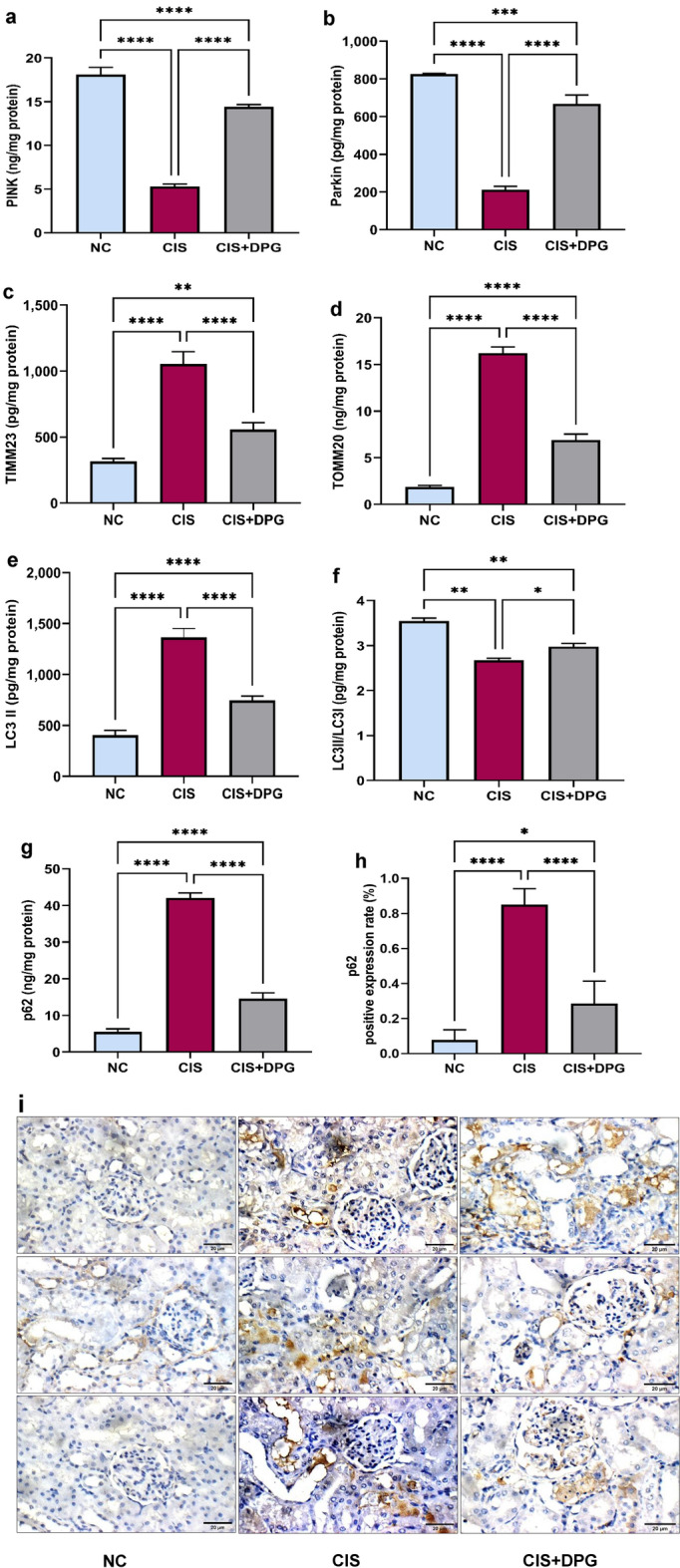
Effects of dapagliflozin pretreatment on mitophagy and autophagy markers in cisplatin-treated rats. The protein expression of (**a**) PTEN-induced kinase 1 (PINK1), (**b**) Parkin, (**c**) Translocase of inner mitochondrial membrane 23 (TIMM23), (**d**) Translocase of outer mitochondrial membrane 20 (TOMM20), (**e**) microtubule-associated protein 1 light chain 3 (LC3)-II, (**f**) LC3II/I ratio and (**g**) p62 in kidney tissues of rats were measured using ELISA. (**h**) Quantification of immunostaining of p62 from the images in (**i**) (× 400), scale bar = 20 µm. All the data were represented as mean ± SD (n = 6). Normal control, NC; Cisplatin, CIS; and Cisplatin + Dapagliflozin, CIS + DPG groups. Statistical analysis was attained using one-way analysis of variance (ANOVA), followed by Tukey–Kramer multiple comparison; *p < 0.05, **p < 0.01, ***p < 0.001, ****p < 0.0001).

Given the crucial role of mitophagy on mitochondrial maintenance, PINK 1, Parkin, TIMM23 and TOMM20 protein levels were assessed using ELISA. CIS administration remarkably reduced protein expression of PINK1, and Parkin by 3- and 4-fold, respectively, as compared to NC rats, suggesting inhibition of mitochondrial clearance. Consistent with the decrease in PINK1 and Parkin levels in CIS-treated rats, TIMM23 and TOMM20 levels were significantly elevated by 3- and 9-fold, respectively, compared to NC rats. Notably, DPG pretreatment caused a significant elevation of PINK 1 and Parkin by 63% and 68%, respectively, alongside a marked decline in TIMM23 and TOMM20 levels by 47% and 57%, respectively, compared to CIS treated rats, suggesting the promising role of DPG in mitophagy regulation (Fig. 5a–d).

### Effects of dapagliflozin pretreatment on autophagy markers in cisplatin-treated rats

CIS-treated rats caused accumulation of LC3-II by 3-fold alongside a marked decrease in LC3-II/LC3-I ratio by 1- fold compared to NC rats. In parallel, p62 levels were clearly increased following CIS administration by 8-fold compared to NC rats, indicating CIS inhibitory effect on autophagic activity. Interestingly, DPG pretreatment mildly but significantly decreased the increment of LC3-II by 45% accompanied by elevation of LC3-II/LC3-I ratio by 11% compared to CIS-treated rats (Fig. 5e,f). In addition, DPG-treated rats exhibited a marked decline in p62 level by 65% compared to CIS treated rats (Fig. 5g). Consistently, pronounced elevation in p62 immunoreactivity was observed in CIS-treated rats to approximately 11-fold compared to NC rats, while DPG pretreatment significantly attenuated p62 immunoreactivity by 66% compared to CIS rats (Fig. 5h,i).

### Effects of dapagliflozin pretreatment on renal histopathological changes in cisplatin-treated rats

**Fig. 6:**
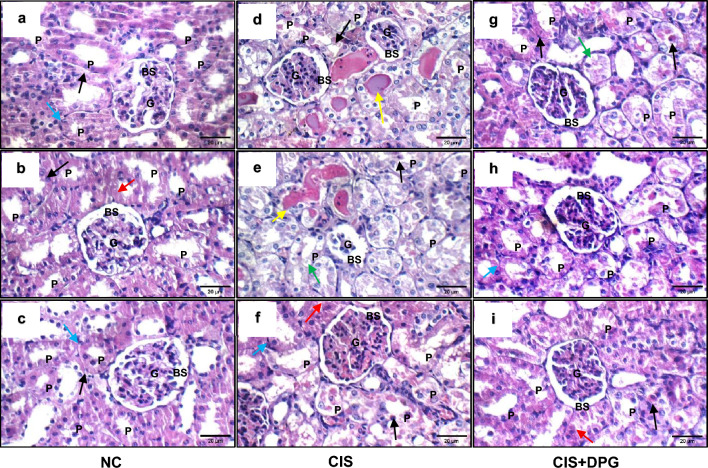
Effects of dapagliflozin pretreatment on cisplatin-induced renal histopathological changes in rats (H&E × 400), scale bar = 20 µm. (**a–c**) Kidney tissues from the normal control (NC) rats showed normal histological features of glomeruli (G), Bowman’s spaces (BS), and proximal tubules (P) with average epithelial lining (black arrow), preserved brush borders (red arrow) and average interstitium (blue arrow). (**d–f**) Cisplatin (CIS) treated rats showed obvious renal injury, as evidenced by congested glomeruli (G), distended Bowman’s spaces (BS), and proximal tubules (P) with marked apoptosis epithelial necrosis (black arrow), edematous epithelial lining (green arrow) and intra-tubular hyaline casts (yellow arrow). (**g–i**) Kidney tissues from dapagliflozin (DPG) pretreated rats exhibited average glomeruli (G) and Bowman’s spaces (BS), restored architecture of tubular epithelial lining with residual epithelial necrosis (black arrow), partial loss of brush borders (red arrow), edematous epithelial lining (green arrow) and average interstitium (blue arrow).

As presented in Fig. 6, kidneys from NC rats displayed normal renal architecture (Fig. 6a–c). In contrast, exposure to CIS provoked acute renal structural changes characterized by renal tubular necrosis, extensive epithelial vacuolations, loss of brush border and hyalin casts formation (Fig. 6d–f). Pretreatment with DPG relatively improved CIS-induced pathological changes in kidneys with only mild swelling of epithelial lining renal tubules, minimal dilation of renal tubules, no detectable alterations in the glomeruli, and minor signs of degeneration with cast formation (Fig. 6g–i). Table 2 shows the effect of DPG on the severity of histopathological alterations in CIS-induced AKI in rats.

**Table 2:** Effects of dapagliflozin (DPG) pretreatment on severity of histopathological changes in in CIS-treated rats.

Histopathological changes	NC	CIS	CIS + DPG
Degeneration	0	+ +	+
Loss of brush border	0	+	+
Dilation	0	+	0
Cast formation	0	+ +	+ +
Renal tubular necrosis	0	+ +	+

AKI scoring was based on assessing degeneration, loss of brush border, dilation, cast formation andrenal tubular necrosis as the following: none (0); mild (+); and moderate (++). (NC) normal control, (CIS) cisplatin,(CIS+DPG) Cisplatin + Dapagliflozin.

AKI scoring was based on assessing degeneration, loss of brush border, dilation, cast formation and renal tubular necrosis as the following: none (0); mild (+); and moderate (+ +). (NC) normal control, (CIS) cisplatin, (CIS + DPG) Cisplatin + Dapagliflozin.

## Discussion

The renoprotective role of SGLT2 inhibitors has been previously documented in animal models of diabetic and non-diabetic kidney diseases through its action against the inflammatory and oxidative status along with apoptosis; even so, the role of SGLT2 inhibitors, particularly DPG on mitophagy regulation in CIS nephrotoxicity has not yet been fully explained^17,26^. This study provided new mechanistic insights regarding the use of DPG in management of CIS-induced nephrotoxicity in rats through restoration of PINK1/Parkin mitophagy pathway besides modulation of ROS/NF-κB and BCL2/Bax signaling pathway.

Pretreatment with DPG ameliorated deterioration of kidney function following CIS administration as verified by reduced levels of SCr and urea nitrogen. The renoprotective role of DPG against CIS insult was further confirmed through mitigation of KIM-1 and NGAL protein expression, early specific biomarkers of kidney damage. Furthermore, DPG treatment suppressed such alterations as depicted by considerable improvement in renal histopathology. CIS administration clearly related to disruption of glomerular filtration, distortion of renal morphology, renal tubular necrosis, and appearance of protein casts as observed in H&E stain herein and previously^31^. In proximal tubular cells, elevation of KIM-1 has been linked to CIS associated AKI, where it probably preceded renal histological changes^32^. While high levels of NGAL following CIS administration were found to be released by neutrophils and epithelial cells of several organs particularly kidney in response to injury^33^. Enhanced KIM-1 and NGAL expression in CIS-treated rats were consistent with results from previous studies^34,35^. In context, studies highlighted the potential therapeutic role of DPG against CIS-induced kidney damage^21,22^.

It is well established that CIS administration is a key driver of excessive ROS production, inflammation and cell death within renal tubular cells^36^. In the present study, CIS-induced oxidative stress is mostly mediated through disruption of redox status where levels of MDA, a lipid peroxidation marker, and NO were progressively elevated with subsequent decline in GSH levels in CIS-treated rats. Nevertheless, treatment with DPG successfully restored the redox status as confirmed by elevating renal level of GSH and lowering expression of MDA and NO, indicating its antioxidant effects. These results corroborated with those of Satyam and co-workers in CIS-induced nephrotoxicity in rats. Treatment with DPG alone or in combination with silymarin was found to remarkably alleviated oxidative stress in CIS-treated rats through restoration of nuclear factor erythroid 2-related factor 2 (Nrf2), transcription factor, and so heme oxygenase-1 (HO-1), pointing to the possible role of DPG as regulator of antioxidant defense against CIS insult^26^.

Beyond excessive ROS production, NF-κB pathway has been considered as potential biomarker related to CIS-induced nephrotoxicity^6^. Alteration in NF-κB levels in CIS-treated rats appeared to boost an intensified state of kidney inflammation resulting in significant elevation of inflammatory mediators, particularly IL-6 and TNF-α. Such alterations were presumably related to structural and functional changes in the kidney. Notably, pretreatment with DPG displayed anti-inflammatory effect, as proven by a marked decline in NF-κB, together with subsequent reduction in IL-6 and TNF-α levels. Similar suppressions were attained by DPG in different experimental models. In earlier study, the anti-inflammatory actions of DPG were explicitly validated in an in vitro model via modulation of NF-κB/NLRP3 inflammasome signaling pathway. It was documented that DPG treatment decreased NLRP3, IL-1β as well as TNF-α levels secondary to suppression of NF-κB nuclear translocation in HK cells treated with high glucose^23^. Moreover, DPG treatment has been shown to mitigate renal injury against LPS insult through downregulation of NF-κB, and downstream cytokines including IL-6 and IL-1 β in diabetic mice^37^.

The present study sheds light on how DPG hindered the deleterious actions of CIS on renal oxidative damage, inflammatory response and apoptosis. Excessive production of ROS and inflammatory mediators, particularly TNF-α were likely associated with CIS-induced mitochondrial dysfunction and exacerbation of tubular injury and cell death. DPG treatment clearly counteracted CIS-induced mitochondrial damage through upregulation of BCL2 gene expression, along with downregulation of renal Bax, pointing to its anti-apoptotic activity. Likewise, the immunoreactivity of cleaved caspase-3, an index of apoptotic pathway, was significantly prevented following DPG pretreatment, reflecting inhibitory effect on mitochondrial apoptotic pathway. Concomitant decline in apoptotic markers such as Bax with restoration of BCL2 level, anti-apoptotic marker, following DPG treatment corroborate with the findings noted by Chang et al. in hypoxic HK2 cells and IR-injured mice models^20^. Moreover, the observed limitation of caspase-3 activation is consistent with DPG inhibitory actions on apoptosis in gentamycin-induced AKI in rat^22^. Of particular importance, the pronounced increase in BCL2 mRNA level in CIS-treated rats compared to NC group suggested an adaptive response against CIS-mediated mitochondrial stress. Nevertheless, BCL2 mRNA level is still lower than in DPG-treated group while Bax gene expression and cleaved caspase-3 immunoreactivity were significantly elevated in CIS-treated rats, suggesting ongoing pro-apoptotic activity. The discrepancy between BCL2 and Bax gene expression emphasizes that the balance between apoptotic and antiapoptotic markers fall short of mitigating mitochondrial mediated cell death in CIS-induced nephrotoxicity.

In fact, the disruption of redox status and mediation of inflammatory response appears to induce mitochondrial-dependent apoptosis, providing insights into mitophagy/autophagy regulation^14^. Activation of PINK1/Parkin pathway, mitophagy dependent pathway, is believed to act as a possible adaptive response for removal of dysfunctional mitochondria^13^. Upon mitochondrial depolarization, PINK1 protein import into mitochondria *via* TIMM/TOMM complexes is disrupted promoting the recruitment of Parkin as well as autophagy machinery like p62 and the activation of mitophagy for removal of damaged mitochondria^16^. In the current study, alteration in PINK1/Parkin signaling pathway in CIS-induced nephrotoxicity was closely related to mitophagy modulation. The results exhibited that CIS-induced renal damage negatively affected PINK1/Parkin signaling pathway causing elevation of mitochondrial proteins TIMM23 and TOMM 20 with substantial decrease in protein expression of PINK1 and Parkin, suggesting impaired mitochondrial clearance. Reducing PINK1 and parkin expression probably suggested that CIS administration impaired mitophagic flux rather than mitophagy activation. In addition, the subsequent analysis showed that CIS administration markedly caused inhibition of autophagic flux as proven by the increment of both p62 and LC3-II along with reduction of LC3-II/LC3-I ratio. Despite the marked decline in PINK1 and parkin, accumulation of LC3-II alongside p62 elevation clearly suggests the notion of impaired autophagic degradation rather than autophagy activation. Collectively, these findings likely support the blockage of autophagic flux alongside compromised mitophagy, highlighting the critical role of PINK/Parkin pathway in clearance of damaged mitochondria and mitochondrial dysfunction. Conversely, a study by Wang and co-workers documented that CIS administration has been shown to inhibit mitophagy with concomitant increase in LC3-II and a marked decrease in p62, suggesting its modulatory effect on autophagic flux^16^. Studies clearly support the fact that CIS role in autophagy regulation is controversial^38,39^. Interestingly, pretreatment with DPG conceivably ameliorated CIS-induced renal tubular injury as verified by elevation of PINK1/Parkin and reduction of TIMM23, TOMM20, p62 and LC3-II abolishing CIS inhibitory effect on mitophagy pathway and autophagic flux. As mitophagy/autophagy activation contributes to proteasomal degradation of p62 through interaction with LC3, these findings probably suggest that DPG may restore autophagic flux disrupted by CIS rather than stimulation of autophagosome formation. In this setting, the regulatory effect of DPG on mitophagy regulation was previously elucidated in type 4 cardiorenal syndrome in vivo model *via* regulation of FUNDC1-dependent mitophagy, as indicated by Shen et al.^40^. Moreover, DPG confers cardiprotective effect in myocardial ischemia reperfusion model by enhancing mitophagy mainly through AMPK-PINK1-dependent pathway^24^. DPG intervention was found to protect against diabetic nephropathy in both in vitro and in vivo models, *via* activation of mitophagy and restoration of mitochondrial functions. DPG promoted mitophagy dependent pathway through upregulation of Sirt1, PINK1, Parkin and BNIP3^25^.Additionally, DPG regulatory role in mitophagy was established against hypothyroidism mediated liver and lung dysfunction model through downregulation of PINK1 and restoration of mitochondrial dynamics^41^.

In summary, the current study demonstrated that pretreatment of DPG could prevent CIS nephrotoxicity *via* activation of mitophagy and autophagic flux. Mechanistically, DPG promotes mitophagy activation through PINK/Parkin pathway. DPG modulatory effects on mitophagy together with oxidative stress, inflammatory response and apoptosis appear to be a potential candidate for maintaining mitochondrial homeostasis and cell survival in AKI. Thus, targeting multiple mechanisms is pivotal in prevention/alleviation of CIS-induced renal injury. Although DPG could be considered as a renoprotective drug, further research incorporating in vitro experiments is warranted to further clarify the direct molecular mechanisms involved in CIS nephrotoxicity, validate its role as a possible mitophagy regulator and establish its beneficial effects in diabetic patients.

## Data Availability

All data generated or analyzed during the current study are listed in the article.

## References

[1] Makris, K. & Spanou, L. Acute kidney injury: Definition, pathophysiology and clinical phenotypes. *Clin. Biochem. Rev.* **37**, 85–98 (2016). 28303073 PMC5198510

[2] Niculae, A. et al. Pathway from acute kidney injury to chronic kidney disease: Molecules involved in renal fibrosis. *Int. J. Mol. Sci.* **24**, 14019 (2023). 37762322 10.3390/ijms241814019 PMC10531003

[3] Kung, C. W. & Chou, Y. H. Acute kidney disease: An overview of the epidemiology, pathophysiology, and management. *Kidney Res. Clin. Pract.* **42**, 686–699 (2023). 37165615 10.23876/j.krcp.23.001 PMC10698062

[4] Gameiro, J., Fonseca, J. A., Outerelo, C. & Lopes, J. A. Acute kidney injury: From diagnosis to prevention and treatment strategies. *J. Clin. Med.* **9**, 1704 (2020). 32498340 10.3390/jcm9061704 PMC7357116

[5] Abd Rashid, N. et al. The role of natural antioxidants in cisplatin-induced hepatotoxicity. *Biomed. Pharmacother.* **144**, 112328 (2021). 34653753 10.1016/j.biopha.2021.112328

[6] Domingo, I. K., Latif, A. & Bhavsar, A. P. Pro-inflammatory signalling PRRopels cisplatin-induced toxicity. *Int. J. Mol. Sci.* **23**, 7227 (2022). 35806229 10.3390/ijms23137227 PMC9266867

[7] Volarevic, V. et al. Molecular mechanisms of cisplatin-induced nephrotoxicity: A balance on the knife edge between renoprotection and tumor toxicity. *J. Biomed. Sci.* **26**, 25 (2019). 30866950 10.1186/s12929-019-0518-9 PMC6417243

[8] Karasawa, T. & Steyger, P. S. An integrated view of cisplatin-induced nephrotoxicity and ototoxicity. *Toxicol. Lett.* **237**, 219–227 (2015). 26101797 10.1016/j.toxlet.2015.06.012 PMC4516600

[9] Dugbartey, G. J. et al. Chemoprotective mechanism of sodium thiosulfate against cisplatin-induced nephrotoxicity is via renal hydrogen sulfide, arginine/cAMP and NO/cGMP signaling pathways. *Int. J. Mol. Sci.* **26**, 384 (2025). 39796237 10.3390/ijms26010384 PMC11720986

[10] Liu, C., Zhou, S., Bai, W., Shi, L. & Li, X. Protective effect of food derived nutrients on cisplatin nephrotoxicity and its mechanism. *Food Funct.* **13**, 4839–4860 (2022). 35416186 10.1039/d1fo04391a

[11] Gómez-Virgilio, L. et al. Autophagy: A key regulator of homeostasis and disease: An overview of molecular mechanisms and modulators. *Cells* **11**, 2262 (2022). 35892559 10.3390/cells11152262 PMC9329718

[12] Alassaf, N. & Attia, H. Autophagy and necroptosis in cisplatin-induced acute kidney injury: Recent advances regarding their role and therapeutic potential. *Front. Pharmacol.* **14**, 1103062 (2023). 36794281 10.3389/fphar.2023.1103062 PMC9922871

[13] Fan, X. et al. *Mitophagy Regulates Kidney Diseases. Kidney Dis.* **10**, 573–587 (2024). 10.1159/000541486 PMC11631111 39664332

[14] Su, L., Zhang, J., Gomez, H., Kellum, J. A. & Peng, Z. Mitochondria ROS and mitophagy in acute kidney injury. *Autophagy* **19**, 401–414 (2023). 35678504 10.1080/15548627.2022.2084862 PMC9851232

[15] Wang, Y., Cai, J., Tang, C. & Dong, Z. Mitophagy in acute kidney injury and kidney repair. *Cells* **9**, 338 (2020). 32024113 10.3390/cells9020338 PMC7072358

[16] Wang, H. et al. Nitrate attenuates cisplatin-induced acute kidney injury by promotion of mitophagy and reduction of oxidative stress. *Curr. Med.* **2**, 8 (2023).

[17] Guo, C. et al. Empagliflozin attenuates renal damage in diabetic nephropathy by modulating mitochondrial quality control via Prdx3-PINK1 pathway. *Biochem. Pharmacol.* **235**, 116821 (2025). 39983849 10.1016/j.bcp.2025.116821

[18] Dhillon, S. Dapagliflozin: A review in type 2 diabetes. *Drugs* **79**, 1135–1146 (2019). 31236801 10.1007/s40265-019-01148-3 PMC6879440

[19] McMurray, J. J. V. et al. Dapagliflozin in Patients with Heart Failure and Reduced Ejection Fraction. *N. Engl. J. Med.* **381**, 1995–2008 (2019). 31535829 10.1056/NEJMoa1911303

[20] Chang, Y. K. et al. Dapagliflozin, SGLT2 inhibitor, attenuates renal ischemia-reperfusion injury. *PLoS. One* **11**, e0158810 (2016). 27391020 10.1371/journal.pone.0158810 PMC4938401

[21] Oraby, M. A., El-yamany, M. F., Safar, M. M., Assaf, N. & Ghoneim, H. A. Dapagli fl ozin attenuates early markers of diabetic nephropathy in fructose- Streptozotocin-induced diabetes in rats. *Biomed. Pharmacother.* **109**, 910–920 (2019). 30551545 10.1016/j.biopha.2018.10.100

[22] Mohamed, D. I., Khairy, E., Saad, S. S. T., Habib, E. K. & Hamouda, M. A. Potential protective effects of Dapagliflozin in Gentamicin induced nephrotoxicity rat model via modulation of apoptosis associated miRNAs. *Gene* **707**, 198–204 (2019). 31075409 10.1016/j.gene.2019.05.009

[23] Xu, J., Kitada, M., Ogura, Y., Liu, H. & Koya, D. Dapagliflozin restores impaired autophagy and suppresses inflammation in high glucose-treated HK-2 cells. *Cells* **10**, 1457 (2021). 34200774 10.3390/cells10061457 PMC8230404

[24] Zuo, W. et al. Dapagliflozin alleviates myocardial ischaemia reperfusion injury by activating mitophagy via the AMPK-PINK1/Parkin signalling pathway. *Curr. Vasc. Pharmacol.* **22**, 203–217 (2024). 38141195 10.2174/0115701611269801231211104905

[25] Ma, Y. et al. SIRT1-PINK1-Parkin axis orchestrated mitophagy and renal repair by dapagliflozin in diabetic nephropathy. *Biochimica et Biophysica Acta (BBA)* **1872**, 168074 (2026). 10.1016/j.bbadis.2025.168074 41106506

[26] Satyam, S. M. et al. Dapagliflozin and silymarin ameliorate cisplatin-induced nephrotoxicity via Nrf2 / HO-1 upregulation : A preclinical mechanistic study. *Sci* **7**, 59 (2025).

[27] Fahmy, M. I., Khalaf, S. S., Yassen, N. N. & Sayed, R. H. Nicorandil attenuates cisplatin-induced acute kidney injury in rats via activation of PI3K/AKT/mTOR signaling cascade and inhibition of autophagy. *Int. Immunopharmacol.* **127**, 111457 (2024). 38160566 10.1016/j.intimp.2023.111457

[28] Livak, K. J. & Schmittgen, T. D. Analysis of relative gene expression data using real- time quantitative PCR and the 2(-Delta Delta C(T)) method. *Methods* **25**, 402–408 (2001). 11846609 10.1006/meth.2001.1262

[29] Abd El-Rhman, R. H., El-Naga, R. N., Gad, A. M., Tadros, M. G. & Hassaneen, S. K. Dibenzazepine attenuates against cisplatin-induced nephrotoxicity in rats: Involvement of NOTCH pathway. *Front. Pharmacol.* **11**, 567852 (2020). 33381027 10.3389/fphar.2020.567852 PMC7768080

[30] Hsu, S. M., Raine, L. & Fanger, H. Use of avidin-biotin-peroxidase complex (ABC) in immunoperoxidase techniques: A comparison between ABC and unlabeled antibody (PAP) procedures. *J. Histochem. Cytochem. Off. J. Histochem. Soc.* **29**, 577–580 (1981). 10.1177/29.4.6166661 6166661

[31] Zhou, L. et al. PINK1 deficiency ameliorates cisplatin-induced acute kidney injury in rats. *Front. Physiol.* **10**, 1225 (2019). 31607953 10.3389/fphys.2019.01225 PMC6773839

[32] Tanase, D. M. et al. The predictive role of the biomarker kidney molecule-1 (KIM-1) in acute kidney injury (AKI) cisplatin-induced nephrotoxicity. *Int. J. Mol. Sci.* **20**, 5238 (2019). 31652595 10.3390/ijms20205238 PMC6834366

[33] Luo, Q. H. et al. KIM-1 and NGAL as biomarkers of nephrotoxicity induced by gentamicin in rats. *Mol. Cell. Biochem.* **397**, 53–60 (2014). 25087119 10.1007/s11010-014-2171-7

[34] Jana, S. et al. Early diagnostic biomarkers for acute kidney injury using Cisplatin-induced nephrotoxicity in rat model. *Curr. Res. Toxicol.* **5**, 100135 (2023). 38033659 10.1016/j.crtox.2023.100135 PMC10682538

[35] Liu, J. et al. Mechanisms of Cisplatin-induced acute kidney injury: The role of NRF2 in mitochondrial dysfunction and metabolic reprogramming. *Antioxidants* **14**, 775 (2025). 40722880 10.3390/antiox14070775 PMC12291884

[36] Elmorsy, E. A. et al. Advances in understanding Cisplatin-induced toxicity: Molecular mechanisms and protective strategies. *Eur. J. Pharm. Sci.* **203**, 106939 (2024). 39423903 10.1016/j.ejps.2024.106939

[37] Chi, P. J., Lee, C. J., Hsieh, Y. J., Lu, C. W. & Hsu, B. G. Dapagliflozin ameliorates lipopolysaccharide related acute kidney injury in mice with Streptozotocin-induced diabetes mellitus. *Int. J. Med. Sci.* **19**, 729–739 (2022). 35582427 10.7150/ijms.69031 PMC9108401

[38] Liao, W. et al. p62/SQSTM1 protects against Cisplatin-induced oxidative stress in kidneys by mediating the cross talk between autophagy and the Keap1-Nrf2 signalling pathway. *Free Radic. Res.* **53**, 800–814 (2019). 31223046 10.1080/10715762.2019.1635251

[39] Shao, Y. F. et al. Kaempferide ameliorates Cisplatin-induced nephrotoxicity via inhibiting oxidative stress and inducing autophagy. *Acta Pharmacol. Sin.* **44**, 1442–1454 (2023). 36658427 10.1038/s41401-023-01051-4 PMC10310756

[40] Shen, Y. et al. Dapagliflozin protects heart function against type-4 cardiorenal syndrome through activation of PKM2/PP1/FUNDC1-dependent mitophagy. *Int. J. Biol. Macromol.* **250**, 126116 (2023). 37541471 10.1016/j.ijbiomac.2023.126116

[41] Mohammed, M. A. et al. Dapagliflozin attenuates hypothyroidism induced liver and lung dysfunction via regulation of mitophagy and apoptosis. *Mol. Biol. Rep.* **53**, 345 (2026). 41627616 10.1007/s11033-026-11503-9

